# Impact of JAK2V617F Mutational Status on Phenotypic Features in Essential Thrombocythemia and Primary Myelofibrosis

**DOI:** 10.4274/tjh.2014.0136

**Published:** 2016-05-16

**Authors:** İpek Yönal, Aynur Dağlar-Aday, Başak Akadam-Teker, Ceylan Yılmaz, Meliha Nalçacı, Akif Selim Yavuz, Fatma Deniz Sargın

**Affiliations:** 1 İstanbul University İstanbul Faculty of Medicine, Department of Internal Medicine, Division of Hematology, İstanbul, Turkey

**Keywords:** JAK2V617F mutation, Essential thrombocythemia, Primary myelofibrosis

## Abstract

**Objective:**

The JAK2V617F mutation is present in the majority of patients with essential thrombocythemia (ET) and primary myelofibrosis (PMF). The impact of this mutation on disease phenotype in ET and PMF is still a matter of discussion. This study aims to determine whether there are differences in clinical presentation and disease outcome between ET and PMF patients with and without the JAK2V617F mutation.

**Materials and Methods:**

In this single-center study, a total of 184 consecutive Philadelphia-negative chronic myeloproliferative neoplasms, 107 cases of ET and 77 cases of PMF, were genotyped for JAK2V617F mutation using the JAK2 Ipsogen MutaScreen assay, which involves allele-specific polymerase chain reaction.

**Results:**

ET patients positive for JAK2V617F mutation had higher hemoglobin (Hb) and hematocrit (Hct) levels, lower platelet counts, and more prevalent splenomegaly at diagnosis compared to patients negative for the JAK2V617F mutation, but rates of major thrombotic events, arterial thrombosis, and venous thrombosis were comparable between the groups. At presentation, PMF patients with JAK2V617F mutation had significantly higher Hb and Hct levels and leukocyte counts than patients without the mutation. Similar to the findings of ET patients, thromboembolic rates were similar in PMF patients with and without theJAK2V617F mutation. For ET and PMF patients, no difference was observed in rates of death with respect to JAK2V617F mutational status. Moreover, leukemic transformation rate was not different in our PMF patients with and without JAK2V617F mutation.

**Conclusion:**

We conclude that JAK2V617F-mutated ET patients express a polycythemia vera-like phenotype and JAK2V617F mutation in PMF patients is associated with a more pronounced myeloproliferative phenotype.

## Abstract (tr)

**Amaç:**

Esansiyel trombositemi (ET) ve primer miyelofibrozis (PMF) tanılı hastaların büyük çoğunluğunda JAK2V617F mutasyonu bulunmaktadır. ET ve PMF’de bu mutasyonun hastalık fenotipi üzerine etkisi halen tartışılmaktadır. Bu çalışmada, JAK2V617F mutasyonunu taşıyan ve taşımayan ET ve PMF hastalarının başvuru sırasındaki klinik parametreler ve hastalık seyri açısından karşılaştırılması amaçlanmıştır.

**Gereç ve Yöntemler:**

Tek merkezli olan bu çalışmada, 107 ET ve 77 PMF olmak üzere toplam 184 Philadelphia-negatif kronik miyeloproliferatif neoplazili hastada bir allel spesifik polimeraz zincir reaksiyonu olan JAK2 Ipsogen MutaScreen kullanılarak JAK2V617F mutasyonu taranmıştır.

**Bulgular:**

JAK2V617F mutasyonunu taşıyan ET hastalarında, mutasyon bulunmayanlara göre tanı sırasındaki hemoglobin (Hb) ve hematokrit (Hct) düzeyleri anlamlı olarak daha yüksek, trombosit sayısı daha düşük ve splenomegali oranları daha yüksek bulunmuştur. Fakat her iki grup arasında majör trombotik olay, arteriyel tromboz ve venöz tromboz açısından fark saptanmamıştır. JAK2V617F mutasyonu bulunan PMF hastalarında ise mutasyon taşımayan gruba göre başvuru anındaki Hb, Hct ve lökosit değerleri anlamlı olarak daha yüksek saptanmıştır. PMF hastalarında, ET hastalarında olduğu gibi tromboembolik olayların JAK2V617F mutasyonundan bağımsız olduğu görülmüştür. ET ve PMF hastalarında JAK2V617F mutasyonu varlığında ölüm oranında farklılık gözlenmemiştir. Bunun yanında JAK2V617F mutasyonunu taşıyan ve taşımayan PMF hastaları arasında lösemik dönüşüm oranı açısından anlamlı bir fark bulunmamıştır.

**Sonuç:**

Bu çalışmanın sonucunda JAK2V617F mutasyonunu taşıyan ET hastalarında polisitemia vera benzeri fenotipin ortaya çıktığı ve bu mutasyonun varlığında PMF hastalarının daha belirgin bir miyeloproliferatif fenotiple ilişkili olduğu söylenebilir.

## INTRODUCTION

Philadelphia-negative chronic myeloproliferative neoplasms (Ph-negative MPNs) are a heterogeneous group including 3 major diseases: polycythemia vera (PV), essential thrombocythemia (ET), and primary myelofibrosis (PMF). Thrombotic events are the major cause of morbidity and mortality in ET. Other complications include hemorrhage and progression to myelofibrosis or acute myeloid leukemia [1,2]. PMF is characterized by a worse life expectancy and a progressive disease course. The disease presents with classically severe anemia, massive splenomegaly, and acute leukemia [3]. JAK2V617F mutation is present in more than 95% of PV patients and approximately 50%-60% of ET and PMF patients [4]. Several studies investigated the clinical relevance of JAK2V617F mutation in ET and PMF patients [5,6,7,8,9,10]. In ET, overall survival (OS) or leukemia-free survival was found not to be affected by the presence of JAK2V617F mutation, while the influence of JAK2V617F on thrombosis or fibrotic transformation remained less clear [5,7,11,12]. Conflicting results have been reported regarding the impact on OS, leukemic transformation rate, and need for chemotherapy or splenectomy in the presence of JAK2V617F mutation [8,9,10,13]. We previously evaluated the clinical and laboratory correlates in 184 patients with Ph-negative MPNs according to the allele burden of JAK2V617F mutation (unpublished data). Herein, we investigate the usefulness of JAK2V617F mutational status for explaining phenotypic variability using the same group of patients, which includes a relatively large series of Ph-negative MPN patients.

## MATERIALS AND METHODS

A total of 184 consecutive Ph-negative MPN patients, 107 with ET and 77 with PMF, admitted to the Division of Hematology of the İstanbul University İstanbul Medical Faculty from 1995 to 2013 were included in the study. ET and PMF patients were diagnosed based on WHO criteria [14]. Informed consent was obtained from all participants according to the local ethics committee guidelines. Complete clinical history, blood count, lactate dehydrogenase (LDH) level, and thrombotic or hemorrhagic complications were recorded. Spleen longitudinal diameters of ≥130 mm to 160 mm and of ≥160 mm on ultrasound were considered as mild and massive splenomegaly, respectively. A scale of 0-3 was used to grade reticulin fibrosis on bone marrow trephine biopsies [15]. The Dynamic International Prognostic Scoring System (DIPSS) plus was used for risk stratification in PMF [16]. Unfavorable karyotypes in PMF were defined as complex karyotype or sole or 2 abnormalities that included +8, -7/7q-, i(17q), inv(3), -5/5q-, 12p-, or 11q23 rearrangement [17]. Patients were genotyped for the JAK2V617F mutation by JAK2 MutaScreen assay (Ipsogen, Luminy Biotech, Marseille, France), which is a TaqMan allelic discrimination assay that contains fluorescent probes specific for wild-type (617V) and mutant (617F) alleles [18].

### Statistical Analysis

Data were processed using SPSS 16 (SPSS Inc., Chicago, IL, USA). Continuous variables were summarized as mean [standard deviation (SD)]. Chi-square statistics were used to compare categorical variables among the different patient groups categorized according to the JAK2V617F mutational status. Analysis of continuous variables among the groups was performed using the Mann-Whitney U test. A p-value of less than 0.050 was considered to indicate statistical significance; all tests were 2-tailed.

## RESULTS

A total of 184 patients (107 with ET and 77 with PMF) were included. Bone marrow fibrosis was detected in 90.7% (97 in 107) of ET and 100% of PMF patients. In ET patients, the grade of bone marrow fibrosis was scaled as follows: grade 0, 9.3%; grade 1, 62.7%; grade 2, 25.2%; and grade 3, 2.8%. All PMF patients had bone marrow fibrosis (grade 2 in 20.8% and grade 3 in 79.2%).

JAK2V617F mutation was identified in 64 of 107 ET (59.8%) and 58 of 77 PMF (75.3%) patients (p=0.028). Clinical and laboratory correlates of ET patients according to JAK2V617F mutational status are summarized in Tables 1 and 2.

JAK2V617F-positive and -negative ET patients showed no significant differences with respect to sex and age at diagnosis. ET patients with JAK2V617F mutation presented with higher hemoglobin (Hb) and hematocrit (Hct) levels and lower platelet count at diagnosis compared to patients without mutation (p=0.001, p=0.001, and p=0.043, respectively). The leukocyte count and LDH levels were similar for the 2 groups.

The 2 groups showed no significant difference with respect to mean spleen size. However, JAK2V617F-positive ET patients presented with more prevalent splenomegaly at diagnosis compared to patients without the mutation (p=0.044).

ET patients with JAK2V617F mutation showed a higher, albeit not statistically significant, rate of bleeding events compared to the JAK2V617F-negative group (15.6% and 7%, respectively; p=0.298).

ET patients with and without JAK2V617F mutation showed no significant difference with respect to the degree of bone marrow fibrosis, prevalence of hydroxyurea use, and rate of splenectomy. In addition, no significant differences were observed in the use of other medical treatments in any of the categories (p>0.050). Duration of follow-up in patients with and without JAK2V617F mutation was 69.7 months (SD: 63.7) and 70.1 months (SD: 56.9), respectively (p=0.675). During follow-up, 3 of 64 (4.7%) JAK2V617F-positive ET and 2 of 43 (4.7%) JAK2V617F-negative ET patients succumbed to their disease (p=1.000).

Clinical and laboratory parameters of PMF patients classified according to genotype are outlined in Tables 3 and 4.

The rate of female patients was higher in the JAK2V617F-negative group compared to the JAK2V617F-positive group (84.2% and 46.6%, respectively; p=0.009). PMF patients with and without JAK2V617F mutation showed no significant differences with respect to age at diagnosis. At initial diagnosis, PMF patients with the JAK2V617F mutation presented with significantly higher Hb and Hct levels and leukocyte counts compared to those without the mutation (p=0.005, p=0.034, and p=0.046, respectively). Platelet count and LDH level did not differ between the 2 groups.

The mean spleen size showed no significant difference among any of the categories, although PMF patients with JAK2V617F mutation showed a trend towards higher prevalence of massive splenomegaly at diagnosis compared to patients without mutation (p=0.193 and p=0.090, respectively).

JAK2V617F-positive PMF patients showed a trend towards a higher prevalence of bleeding events compared to JAK2V617F-negative PMF patients (24.1% and 5.3%, respectively; p=0.090).

There was no significant difference in the prevalence of total thrombotic events, arterial thrombosis, and venous thrombosis between JAK2V617F-positive and -negative PMF patients.

The degree of reticulin fibrosis, prevalence of hydroxyurea use, rate of allogeneic hematopoietic stem cell transplantation (AHSCT), and history of splenectomy did not differ in any of the categories. In addition, the 2 groups showed no significant differences in the use of other medical treatments (p>0.050).

No significant difference was observed in the distribution of karyotype categories and DIPSS-Plus risk stratification between JAK2V617F-positive and -negative PMF patients.

Duration of follow-up in PMF patients with and without JAK2V617F mutation was 42 months (SD: 46.9) and 56.6 months (SD: 48.7), respectively (p=0.165). At the end of the data collection period, 11 of 58 (19%) PMF patients with JAK2V617F mutation succumbed to their disease, while the rate of death in patients without JAK2V617F mutation was 15.8% (p=1.000). During follow-up, rate of leukemic transformation was similar between the 2 categories.

## DISCUSSION

In our relatively large series of patients with Ph-negative MPNs, including 107 ET patients with a mean follow-up duration of more than 5 years and 77 PMF patients with a mean follow-up duration of more than 3 years, we documented that JAK2V617F mutation correlates with disease phenotype in adult Turkish patients with ET and PMF.

Our results suggest that JAK2V617F positivity in ET induces a phenotype resembling PV. Confirming previous observations, we found that ET patients with JAK2V617F mutation presented with higher Hb and Hct levels and lower platelet counts compared to unmutated patients [5,6,7,19,20,21,22]. Contrary to some previous reports yet consistent with the findings of Kittur et al. [5] and Pich et al. [22], our ET patients with JAK2V617F mutation showed no difference in leukocyte count at diagnosis as opposed to patients without the mutation [6,21]. Furthermore, in contrast to some previous reports but consistent with the study of Vannucchi et al. [11], we observed a higher prevalence of splenomegaly in ET patients with JAK2V617F mutation than in patients without the mutation [5,6,7,20,21]. Data on ET regarding the impact of JAK2V617F mutational status on thrombotic events are conflicting. In the study by Campbell et al., JAK2V617F mutation in ET was associated with an increased frequency of venous thromboembolism, but not with arterial thrombosis [6]. In the study by Kittur et al., the presence of JAK2V617F mutation was found to be significantly associated with increased incidence of venous thrombosis during follow-up, but not with major thrombosis, arterial thrombosis, and venous thrombosis at diagnosis [5]. In contrast, Antonioli et al. reported that there was no correlation between thrombotic events and JAK2V617F mutation in ET patients [20]. In another study, there was no difference between ET patients with JAK2V617F mutation or wild-type alleles with respect to the frequency of major thrombotic events and major arterial and venous thrombosis, either at diagnosis or during follow-up [21]. Similar to the aforementioned study in ET patients, the presence of JAK2V617F mutation made no significant difference in the frequency of vascular complications at presentation [7]. In the current study, we observed no significant difference in the frequency of major thrombotic events, arterial thrombosis, and venous thrombosis between JAK2V617F-positive and -negative ET patients. In the study by Pich et al., ET patients with JAK2V617F mutation were younger than those without mutation [22]. Conversely, in several studies, the presence of JAK2V617F mutation was significantly associated with older age at diagnosis [5,7,11,21,23,24,25]. Some studies revealed no difference in age between JAK2V617F-positive and -negative ET patients [20,26]. In our study group, we found no significant difference in age among ET patients with and without JAK2V617F mutation. Moreover, in the current study, we did not determine an association between JAK2V617F mutation and sex, consistent with previous reports [5,7,11,20,21,23,24,25,26]. Alvarez-Larrán et al. reported that the presence of JAK2V617F mutation in ET patients was associated with increased LDH levels [25]. On the contrary, in another study, JAK2V617F mutation in ET did not correlate with LDH level [21]. Our ET patients with JAK2V617F mutation did not show differences in LDH level as compared to wild-type patients. To our knowledge, there is limited information about the association between JAK2V617F mutation and histological changes in bone marrow biopsy of ET patients. In a series of 103 ET patients, Pich et al. reported no significant impact of JAK2V617F mutation on bone marrow fibrosis [22]. In the current study, the presence of JAK2V617F mutation in ET did not correlate with the degree of reticulin fibrosis. Several studies investigated the association between JAK2V617F mutation in ET and major hemorrhages [7,11,20,21,25]. Confirming the findings of the aforementioned studies, our ET patients with mutant and wild-type alleles showed no differences in the rate of bleeding complications [7,11,20,21,25]. Some previous studies reported that cytoreductive therapy requirement did not differ between ET patients with and without JAK2V617F mutation [7,21,23,24]. This finding is in line with our data showing that the prevalence of hydroxyurea use and other medical treatments was similar between JAK2V617F-mutated and -unmutated ET patients [7,21,23,24]. In ET patients, OS was shown not to be influenced by the presence of JAK2V617F mutation [5,7]. Confirming this observation, the death rate did not differ in our ET patients with and without JAK2V617F mutation.

In our series of 77 PMF patients, we found a significant association between JAK2V617F mutation and the expression of a more pronounced myeloproliferative phenotype. In PMF patients, JAK2V617F mutational status contributed to laboratory abnormalities, including higher Hb level and leukocyte count, but its association with platelet count is inconsistent [19]. Our PMF patients with JAK2V617F mutation had higher Hb and Htc levels and leukocyte counts at diagnosis than those without the mutation. In contrast, in our PMF patients, platelet count at initial diagnosis did not differ with respect to the JAK2V617F mutation. Barosi et al. demonstrated the association between JAK2V617F mutational status and development of marked splenomegaly [9]. On the other hand, in this population, several other groups did not show any correlation between the presence of JAK2V617F mutation and spleen size [8,10]. In the study by Guglielmelli et al., JAK2V617F mutated and wild-type patients did not differ from each other as regards the presence of palpable splenomegaly greater than 15 cm from the left costal margin [27]. In our study, the mean spleen size did not significantly differ between JAK2V617F-positive and -negative PMF patients, although PMF patients with JAK2V617F mutation showed a trend towards higher prevalence of massive splenomegaly at diagnosis compared to patients without mutation. In PMF patients, the relationship of JAK2V617F mutation and thrombosis is controversial. In the study by Barosi et al., there was no significant difference in the rate of major thrombotic events between JAK2V617F-mutated and -unmutated PMF patients [9]. In a series of 199 PMF patients, Tefferi et al. showed no significant difference in the prevalence of thrombosis between JAK2V617F-positive and -negative PMF patients, whereas in another series of 117 PMF patients, Tefferi et al. reported the association of the presence of JAK2V617F mutation with history of thrombosis [8,13]. In the current study, the prevalence of total thrombotic events, arterial thrombosis, and venous thrombosis did not significantly differ among PMF patients with and without JAK2V617F mutation. Several studies have shown that ET patients with mutant alleles and wild-type alleles showed no significant difference with respect to age and sex [8,10,27]. In the current study, the presence of JAK2V617F mutation in PMF patients was not associated with age. However, in our study, the rate of females was higher among JAK2V617F-negative PMF patients than JAK2V617F-positive PMF. We did not find a significant difference in LDH level between PMF patients with and without JAK2V617F mutation, in accordance with some previous reports [8,10,27]. In a study involving 117 patients with PMF, the presence of JAK2V617F mutation did not correlate with degree of reticulin fibrosis [8]. Consistent with the study by Tefferi et al., the degree of reticulin fibrosis did not differ between our PMF patients when stratified by JAK2V617F mutational status [8]. There is limited information regarding the relevance of JAK2V617F on bleeding complications in PMF patients. Tefferi et al. did not determine a statistically significant correlation between JAK2V617F mutation and bleeding history [8]. However, we observed a trend towards higher prevalence of bleeding events in JAK2V617F-positive PMF patients compared to JAK2V617F-negative PMF patients (24.1% and 5.3%, respectively). In the study by Barosi et al., JAK2V617F mutational status was associated with an increased requirement for splenectomy and greater need of cytoreductive therapy in PMF patients [9]. However, in the study by Tefferi et al. involving 199 patients with PMF, no significant correlation was found between the presence of JAK2V617F mutation and need for cytoreductive therapy or splenectomy [13]. Confirming the finding of Tefferi et al., in our study, the presence of JAK2V617F mutation in PMF had no impact on the need for cytoreductive treatment or requirement for splenectomy [13]. Several studies investigated the association of JAK2V617F mutation in PMF patients with prognostic scoring systems [8,10,13,27]. In a series of 186 PMF patients, the number of JAK2V617F-positive patients in the low risk category of the Dupriez scoring system was significantly higher compared with JAK2V617F-negative patients [27]. Campbell et al. reported that Dupriez prognostic scores tended to be lower for patients positive for JAK2V617F mutation [10]. On the contrary, several groups reported no correlation between JAK2V617F mutation and Dupriez prognostic score [8,13]. To analyze whether the JAK2V617F mutational status correlated with prognostic scoring systems, we evaluated the distribution of patients in the different risk categories of the DIPSS-Plus [16]. We found no significant difference in the DIPSS-Plus risk stratification between JAK2V617F-positive and -negative PMF patients. Several studies revealed that in PMF, the presence of JAK2V617F mutation showed no correlation with presence or distribution of cytogenetic abnormalities [8,9,10]. Confirming the aforementioned studies, in our population, we observed no significant difference in the distribution of karyotype categories between JAK2V617F-positive and -negative groups. Divergent results were reported regarding the effect of JAK2V617F mutation on OS and leukemic transformation rate in PMF patients [8,9,10,13]. We did not observe any differences in the rates of death and leukemic transformation in PMF patients with and without JAK2V617F mutation.

Collectively, according to the results of our study, JAK2V617F mutation may identify distinct disease phenotypes of ET and PMF patients.

## Ethics

Ethics Committee Approval: The study was approved by the Local Ethics Commitee of İstanbul University İstanbul Medical Faculty (file number: 2012/1571-1245), Informed Consent: Informed consent was obtained from all patients for being included in the study.

## Figures and Tables

**Table 1:**
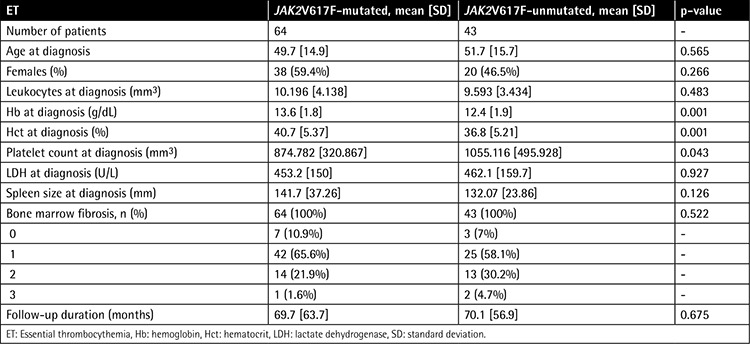
Clinical and laboratory features between JAK2V617F-mutated and -unmutated patients among 107 patients with essential thrombocythemia.

**Table 2:**
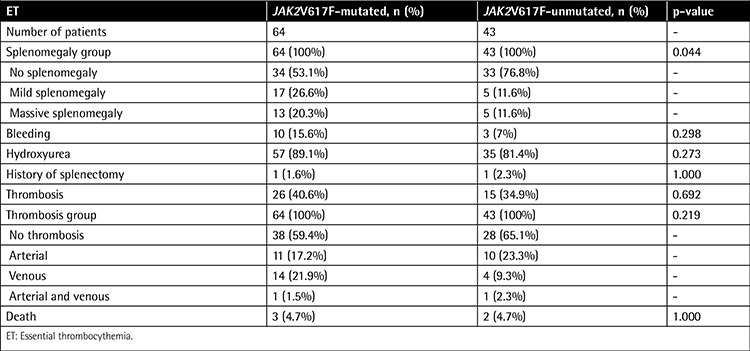
Clinical and laboratory features between JAK2V617F-mutated and -unmutated patients among 107 patients with essential thrombocythemia (continued).

**Table 3:**
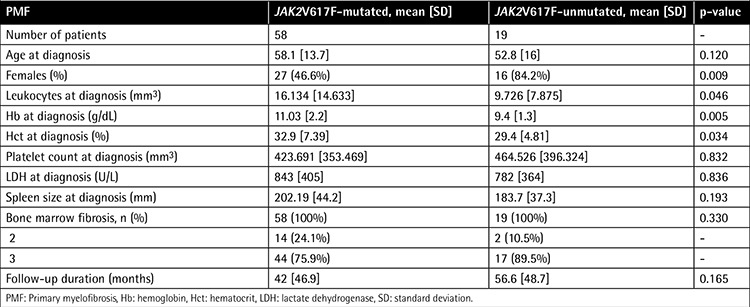
Clinical and laboratory features between JAK2V617F-positive and -negative patients among 77 primary myelofibrosis patients.

**Table 4:**
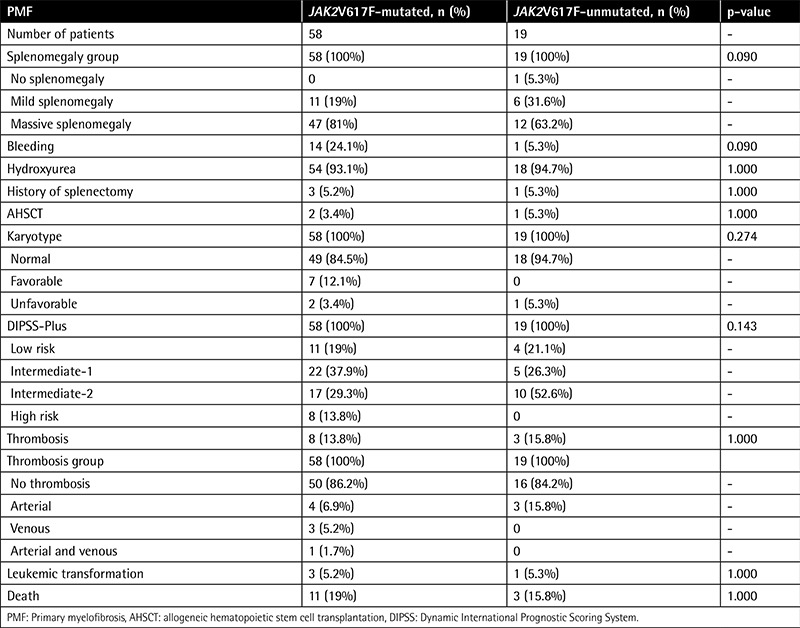
Clinical and laboratory features between JAK2V617F-positive and -negative patients among 77 primary myelofibrosis patients (continued).
